# Knowledge of Oral and Physical Manifestations of Anorexia Nervosa Among Polish Dentists: A Cross-Sectional Study

**DOI:** 10.3389/fpsyt.2021.751564

**Published:** 2021-10-27

**Authors:** Agnieszka Krukowska-Zaorska, Katarzyna Kot, Ewa Marek, Włodzimierz Dura, Krzysztof Safranow, Mariusz Lipski

**Affiliations:** ^1^Department of Preclinical Conservative Dentistry and Preclinical Endodontics, Pomeranian Medical University, Szczecin, Poland; ^2^Department of Biochemistry and Medical Chemistry, Pomeranian Medical University, Szczecin, Poland

**Keywords:** anorexia nervosa, dentists, diagnosis, knowledge, questionnaire

## Abstract

**Background:** Anorexia nervosa is a psychosomatic disorder and is commonly associated with impaired oral health. Dentists can play a relevant role in the early diagnosis of anorexia nervosa. With the help of intra- and extraoral examinations and anamnesis, the dentist can detect characteristic signs and symptoms of this eating disorder. The purpose of this study was to determine the knowledge regarding the oral and physical manifestations of anorexia nervosa among general dental practitioners and specialist dentists of Poland.

**Material and Methods:** A pretested online questionnaire consisting of a first part asking for the characteristics of the participant and a second part with 22 specific questions on their general knowledge of anorexia nervosa and knowledge of physical and oral manifestations of this eating disorder was used.

**Results:** A total of 369 dentists completed the questionnaire. The Polish dentists in this study reported sufficient knowledge regarding anorexia nervosa. However, younger dentists and general dental practitioners had relatively lower knowledge scores than other groups. The dentists mainly had difficulties with the oral symptoms of anorexia nervosa.

**Conclusion:** Despite Polish dentists having sufficient knowledge about anorexia nervosa in relation to the general symptoms of anorexia, there are deficits with regard to oral manifestations. Therefore, there is a need to increase continuing education in this field, which can improve early diagnosis of this disease by dental practitioners and referral to specialists for treatment.

## Introduction

Nowadays, the beauty stereotype imposed by society demands that people be thinner and have nice and well-groomed bodies. This fact can cause a divergence in perception between the idealized body and the real body and may contribute to the subsequent emergence of eating disorders (1).

Among the main eating disorders classified as mental illnesses, we can mention anorexia nervosa (AN), bulimia nervosa, binge eating disorder, and eating disorders not otherwise specified (EDNOS) (2). This research focuses on AN, which is the disease with the highest rate of mortality among all mental disorders and the third most common chronic disease among female adolescents (3–5). While most people with AN return to a normal state of health, about 5% die and 20% develop a chronic eating disorder (6). Suicide is considered the main cause of death but circulatory, metabolic, and/or electrolyte disturbances resulting from extreme cachexia also play a role in the increased mortality (7).

AN is a serious, mental illness related to eating disorders and distorted body images (6–8). The cause of this disorder has been related to family, cultural and psychiatric factors (9, 10). People with AN intentionally refuse to eat or maintain a minimal body weight, consider themselves obese, and have an excessive fear of gaining weight or a desire to lose weight (6–9). They pay attention to controlling their weight and shape by using extreme efforts, and it becomes an obsession in their lives (10–12). They usually avoid food and meals that they perceive as high in calories by eating only a small portion of the food or by carefully weighing and dividing food into portions (13). Over time, systematic fasts appear, carefully hidden from family and friends. Dietary restrictions are a way to help them cope with emotional challenges. The patient's driving force is the noticeable effects of his or her work and the constantly decreasing body weight. In this way, they improve their self-esteem and gain self-satisfaction (13). People with AN check their body weight over and over again. They are embarrassed about their behavior, as they don't want to be caught and their weight loss to be interrupted (14). Anorectics feel uncomfortable when other people eat around them. More often, patients prefer to eat alone or discreetly throw away food. Lack of appetite is explained by malaise, food allergies or ailments from the digestive system (15, 16).

Three diagnostic criteria must be present to establish a diagnosis of AN:

1. Restriction of energy intake relative to requirements, leading to a significantly low body weight.
2. Intense fear of gaining weight or becoming fat.
3. Distorted perception of body weight or shape.

According to the 5th version of the Diagnostic and Statistical Manual of Mental Disorders (DSM-5) a criterion of amenorrhea (absence of menstrual periods) is no longer required to diagnose AN (11). It is important because this criterion caused men to be underrepresented and limited in research about eating disorders (17). It also allows females who have menstruation, despite extreme weight loss and malnutrition, to meet the inclusion criteria.

Two subtypes of AN have been defined: binge-purge and restricting. The first type is characterized by regular binge eating and purging behavior. In the other one, weight loss occurs mainly through caloric restriction and excessive exercise (6, 9, 13).

Various signs and symptoms of systemic diseases can be manifested in the oral cavity (18, 19). Oral symptoms of AN can be seen as early as 6 months after a person is continuously engaged in eating behaviors involving caloric restriction and vomiting (20). In AN, oral manifestations include enamel erosion, the trauma of oral mucosa and pharynx, parotid gland enlargement and xerostomia. Prevalence of dental caries and periodontal diseases among AN remains unclear (21–23). They can be identified in a dental office if only oral and some extraoral manifestations associated with this disease are known (1, 3, 6, 9, 21–23). The dentist's key role is to be the first health care practitioner to recognize, evaluate the oral effects of AN, and make a critical referral of this patient to the appropriate physicians. Failure to identify oral and some extraoral manifestations of AN may reduce the likelihood of early treatment and lead to more serious systemic problems.

There are few studies about the knowledge, attitudes, and clinical experience of dentists in the management of AN in different countries, but there is no data available as to the awareness of AN among Polish dentists (14, 22, 24–26). The aim of this study was to assess the level of knowledge regarding oral and physical manifestations of AN among Polish dentists.

## Materials and Methods

### Study Design

This cross-sectional study was performed among Polish dentists. The sampling method was based on the convenience technique, which is speedy, easy, readily available, and possible during the Covid-19 epidemic.The main instrument to collect data was an online anonymous questionnaire using Google forms. A brief survey was designed to measure knowledge of dentists with regard to the general and oral health perspectives of AN. The questionnaire was delivered during April and May 2021. The link to the survey was sent by e-mails, researchers themselves contacted dentists to participate in the study, and participating dentists were also asked to forward the questionnaire to their colleagues to achieve maximal participation. The link to the questionnaire was also posted on social media (Facebook groups including “Dentists,” “Dentists–Cases,” “Courses,” and “Discussions”).

### Questionnaire

The questionnaire items were developed based on DSM-5 diagnostic criteria (American Psychiatric Association, 2013) and previous findings from the literature review (14, 22, 24–26). The model of the survey for dentists was created by the authors. The questionnaire was divided into three parts and had a total of 31 questions. Upon clicking on the link, there was an explanation of the purpose of the study and the voluntary and anonymous nature of the survey.

The first part contained demographic characteristics (gender, age, specialization, region of practice [village or cities], years of practice, and mean number of patients per week −9 questions) and general information about the level of knowledge about AN (6 questions), whereas the second and third parts assessed the knowledge of physical manifestations of AN (five questions) and the stage of knowledge about oral manifestations of AN (11 questions). The questions were closed questions, with each item offering four response choices—“agree,” “disagree,” “don't know,” or “unsure–among researchers there are different opinions.” The questionnaire was written in the Polish language. Before distributing surveys to dentists, a pilot study was conducted among 20 dentists (members from the Faculty of Medicine and Dentistry, Pomeranian Medical University, and dentists practicing in Szczecin). These dentists judged whether the instrument was clear and understood. Then, the questionnaire was modified according to suggestions. The inclusion criteria were the general dentists or specialists from public and private practices from Poland with Internet access. The pilot study responses, incomplete responses (those with greater than or equal to one missing answer), and those who respond after the date Mai 31, 2021 were excluded from the main analysis. Custom Microsoft Office Excel formulas were used for automatic storage, scale, and scored the participants' responses.

### Sample Size Calculation

The sample size was calculated using the online Raosoft sample size calculator explicitly designed for population surveys (www.roasoft.com/samplesize.html). Assuming 38,593 dentists are actively practicing dentists (the license registry data of the General Medical Chamber, on October 5, 2020), with the total number of respondents being 369 and the confidence level of 95%, the error margin was 5.1%.

### Ethical Approval

The study was registered by the local Ethical Committee of the Pomeranian Medical University in Szczecin (Szczecin, Poland) (KB −0012/89/06/22021/Z). No separate ethical approval was necessary.

### Statistical Analysis

Descriptive statistics were calculated regarding gender, age, specialization, region of practice, years of practice, and mean number of patients per week. For each question, survey responses were summarized using percentages. The dentists' knowledge was considered poor if <60% of the respondents answered the question correctly. Moreover, the number of correct answers was summed up for each respondent; 1 point was awarded for a correct answer, 0 point for an incorrect answer. The distributions of responses rated to a two-point scale (correct answer −1 point; incorrect answer −0 point) were compared between groups using the Kruskal-Wallis test and Siegel Castellan *post hoc* test. The significance level was considered to be *p* < 0.05. Statistical analyses were performed using Statistica 13 (Statsoft, Tulsa, OK, USA).

## Results

### Characteristics of the Study Participants

A response was received from 369 dentists. The majority of participants were female (83.2%), and dentists practiced in cities with more than 200,000 inhabitants (70.5%). In addition, 158 individuals were dentists with specialization and 211 individuals were general practitioners. Table 1 demonstrates demographic data, such as age, specialization, location of practice, years of practice, and mean number of patients per week.

**Table 1:** Demographic characteristics of the study participants.

**Variable**		***N***	**(%)**
Gender			
	Female	307	83.2
	Male	62	16.8
Age (years)			
	24–30	94	25.5
	31–40	135	36.6
	41–50	44	11.9
	51–60	91	24.7
	≥60	5	1.4
Clinical experience (years)			
	≤ 3	54	14.6
	4–10	96	26.0
	11–20	106	28.7
	≥21	113	30.6
Designation			
	General practitioner	211	57.2
	Specialist	158	42.8
Location of practice			
	Village	20	5.4
	City <50,000 inhabitants	32	8.7
	City 50,000–200,000 inhabitants	57	15.4
	City > 200,000 inhabitants	260	70.5
Number of patients per week			
	≤ 15	65	17.6
	16–30	125	33.9
	31–45	90	24.4
	≥46	89	24.1

### Dentist's Knowledge

The maximum possible score for knowledge questions was 22, and individual scores were normally distributed, ranking from 4 to 22 (mean = 15.4). There were no statistically significant differences in knowledge scores in relation to gender and the number of patients treated per week. However, the age and clinical experience of dentists influenced their knowledge. The level of knowledge initially declined, then increased in the older and with more clinical experience. Compared to general practitioners and respondents practicing in rural areas, specialists and dentists practicing in large cities (over 200,000 inhabitants) had significantly greater knowledge (Table 2).

**Table 2:** Knowledge scores in different demographic characteristics.

**Variable**		**Score**
Gender		
	Female	15.4
	Male	15.2
Age (years)		
	24–30	15.5
	31–40	14.5ab
	41–50	16.3a
	51–60	16.0b
	≥60	19.0
Clinical experience (years)		
	≤ 3	15.2
	4–10	14.8a
	11–20	15.2
	≥21	16.2a
Designation		
	General practitioner	14.8a
	Specialist	16.3a
Location of practice		
	Village	13.7a
	City <50,000 inhabitants	14.8
	City 50,000–200,000 inhabitants	14.8
	City > 200,000 inhabitants	15.7a
Number of patients per week		
	≤ 15	16.6
	16–30	14.8
	31–45	15.1
	≥46	15.6

*The same small letters represent statistically significant differences at p < 0.05 in the same column (Kruskal-Wallis test)*.

Questions and frequency distributions of correct answers in percentage are reported in Table 3. The majority of dentists correctly identified people with AN that had low body weight and that had hormonal disorders manifested by the disappearance of menstruation, as well as untreated anorexia can lead to death in extreme cases (94.9, 98.6, and 99.2%, respectively). In all, 95.9% of respondents agreed that AN can change skin conditions to dry, rough, and covered with lanugo-like body hair, while only 52.8% of respondents knew that AN is curable diseases. To sum up, for only one question, the percentage of correct answers was lower than 60.

**Table 3:** Questions about knowledge of physical and oral manifestations of anorexia nervosa (AN).

**Questions**	**Distributions of answers (%)**			
	**Agree**	**Disagree**	**Don‘t know**	**Unsure**
Anorexia occurs with a similar frequency in both females and males	7.9	**81.8**	5.1	5.1
Young people in adolescence suffer from anorexia more often than adults	**83.5**	7.6	4.9	4.1
People with anorexia have a low body weight	**94.9**	1.4	0.0	3.8
People with anorexia tend to eat alone	**64.2**	20.1	14.9	0.8
People with anorexia have hormonal disorders manifested by the disappearance of menstruation	**98.6**	1.4	0.0	0.0
Untreated anorexia can lead to death in extreme cases	**99.2**	0.8	0.0	0.0
Anorexia is considered an incurable disease	16.8	**52.8**	14.4	16.0
The skin of patients suffering from anorexia can be rough, dry, and covered with a characteristic lanugo-like body hair	**80.8**	19.2	0.0	0.0
In patients with anorexia, bruises, abrasions, calluses may be visible on the dorsal of the hands	**65.6**	4.6	29.0	0.8
Due to vitamin deficiencies, the hair of patients with anorexia nervosa is brittle and falls out	**95.9**	0.0	4.1	0.0
Patients with anorexia happen erosions and inflammation of the nail	**80.2**	3.0	15.9	0.8
A common extraoral symptom of anorexia is a painless enlargement of the parotid glands	**42.3**	6.0	51.8	0.0
In the course of anorexia, teeth hypersensitivity to cold, heat, mechanical stimuli, e.g., brushing, is often observed	**71.6**	9.5	18.2	0.8
For people with anorexia, an increase in caries development is often observed	61.0	19.5	15.7	3.8*
In patients with anorexia, deep periodontal disease is more often observed	64.2	8.1	22.5	5.1*
In patients with anorexia can be seen the loss of tooth structure erosion	**68.8**	22.8	6.8	1.6
Enamel erosion occurs on the lingual and occlusal surfaces of the maxillary lateral teeth	**67.5**	22.0	8.9	1.6
Enamel erosion occurs on the lingual surfaces of the anterior teeth in the mandible	**42.3**	41.2	14.9	1.6
Enamel erosion occurs on the labial surfaces of the front teeth	35.2	**52.0**	9.8	3.0
Patients with anorexia may complain of xerostomia	**84.3**	0.0	14.9	0.8
In the course of anorexia may appear the inflammation of the tongue and cheilitis angularis	**93.8**	1.1	5.1	0.0
In the mucosa of the soft palate in patients with anorexia injury, bruises, abrasions may occur	**55.8**	17.9	24.7	1.6

*The correct answers are marked in bold.*

*Literature inconsistent with regard to dental caries and periodontal disease as manifestations of AN; thus, correct answer would be “unsure”.

For some questions about oral manifestations of AN, the dentists did not have any problems with the correct answers as follows: 93.8% of responding dentists correctly identified inflammation of the tongue and cheilitis angularis, 84.3% of dentists knew that xerostomia can appear in patients with AN, and 68.8% of dentists considered that AN leads to loss of tooth enamel as a consequence of erosion. However, only 3.8 and 5.1 % (respectively) of respondents correctly answered the question about the increased incidence of caries and deep periodontal disease in patients with AN. Every second respondent gave the correct answer to the question about the likelihood of erosion in the front teeth, and 42.3% of dentists knew that painless enlargement of the parotid glands is a common extraoral symptom of AN. To sum up, in the case of six out of 11 questions concerning oral manifestations of AN, the percentages of correct answers given by the respondents were lower than 60.

### Self-Rated Knowledge

Self-rated knowledge was in agreement with assessed knowledge. The scores ranged from 13.3 (poor self-rated knowledge) to 16.0 (very good self-rated knowledge) (Table 4).

**Table 4:** Self-rated knowledge about anorexia nervosa among dentists.

**Acquired**	***N***	**(%)**	**Score**
Poor	38	10.3	13.3a
Sufficient	144	39.0	15.3
Good	167	45.3	15.9a
Very good	20	5.4	16.0

*The same small letters represent statistically significant differences at p < 0.05 in the same column (Siegel Castellan post hoc test)*.

### Sources of Knowledge

Table 5 presents the sources of information utilized by participants regarding AN. The majority of dentists reported the media (62.7%) as the main source and a considerable percent depended on self-studies (48.5%) and dental school (42.5%), respectively.

**Table 5:** Sources of acquired knowledge regarding anorexia nervosa (more than one response alternative was allowed).

**Sources of acquired knowledge**	***N***	**(%)**
Media	231	62.6
Own experience	118	32.0
Dental school	157	42.5
Self-studies	179	48.5
Courses	38	10.3
Other sources	41	11.1

## Discussion

The pursuit of your dream figure or being unable to cope with problems may lead to the development of eating disorders. AN is one of the eating disorders, causing serious somatic effects that threaten the health and even life of girls and young women. The highest incidence of AN is between 17.1 and 20.8 years of age. In all, 90–95% of anorexics are under 25 years of age (21). This phenomenon may be explained by the greater influence of the media on young women, who show a higher level of internalization of cultural patterns in the body shape than men. Women's magazines and programs especially create certain canons of beauty, such as a slim body. Currently, little epidemiological studies on AN have been conducted in Poland (27–29). It is estimated that, among girls below 18 years of age in Poland, the problem of AN affects 0.8–1.8%, and after taking into account the two different types, its prevalence is as high as 3.7% (28). AN is more often observed in females, but the morbidity of men was higher and ranged between 0.1 and 0.3% (29). The reason for this situation is that males often receive a late diagnosis due to the misconceptions that AN does not affect them.

The purpose of our study was to obtain the stage of knowledge about general, physical, and oral manifestations of AN among Polish dentists. To the best of our knowledge, this is the first cross-sectional survey conducted to assess the knowledge of dental practitioners about AN in Poland. Our results showed that dentists' awareness about AN is sufficient in relation to the general symptoms of anorexia. However, some gaps in knowledge regarding oral manifestations were identified.

The dentists had no difficulty answering questions on general knowledge of AN, although half of the respondents knew it was curable and two out of three dentists knew that people with AN tended to eat alone. Treatment of AN is difficult and is carried out by multidisciplinary teams (individual, family, and group therapy; nutritional and psychoeducation; and behavior therapy to normalize eating behavior) (30). A recent study of primary care in patients with AN showed that two-thirds achieved clinical recovery within 5 years (31). Dentists also responded pretty well to questions about physical manifestations (hair loss, erosive inflammation of the nails, dryness of skin, and lanugo). The most difficult question was the question about the presence of the calluses, abrasions, and bruises on the dorsal surface of the hand (two out of three dentists answered correctly). Callus formation on the knuckles or dorsal surface of the hand is known as Russell's sign and is characteristic of self-induced vomiting anorectics (10, 21).

On the other hand, the results of our study show that dentists have insufficient knowledge of oral manifestations. A relatively low percentage of correct answers was given to the enlargement of the parotid glands (42.3%), which is an often extraoral symptom of anorexia (9, 32, 33). The etiology of parotid enlargement has not been explained. In patients with a self-reported frequency of binge eating and self-induced vomiting, a painless uni- or bilateral enlargement deforming facial features was observed (34, 35). Cholinergic stimulation of the glands during vomiting and autonomic stimulation of the glands by activating the taste buds are two mechanisms explaining the enlargement of the salivary glands (36). The enlargement of the salivary glands is accompanied by reduced salivation, which leads to the xerostomia (9, 37). Drugs administrated during the treatment of patients with diagnosed AN, as well as a dehydration caused by episodes of vomiting, contribute to xerostomia (13). On the other hand, 84.3% of respondents correctly answered the question that patients with AN may complain of xerostomia.

Dry mouth, poor oral hygiene, specific diet, and xerostomia are the factors that predispose to tooth decay and gingivitis (9, 22, 32). Reports about dental caries in people with anorexia are contradictory. The differences in the prevalence of caries resulting from disordered eating may arise from an individual's oral hygiene, the cariogenicity of the diet, malnutrition, genetic predisposition, exposure to fluoride during tooth development, and ingestion of certain types of medication (22). Patients with AN often have a higher consumption of carbonated, acidic, or caffeinated beverages; citrus fruits; and high-carbohydrate foods. Poor oral hygiene is common in AN, especially when associated with depression (9). In contrast, some patients may exhibit a high level of oral hygiene, especially with compulsive oral hygiene practice (38). Hellstrom's (39) study about caries development in patients with eating disorders showed there were no differences between vomiters and non-vomiters. These findings were not corroborated by Hurst et al. (40) and Philipp et al. (34) who reported that patients who were non-vomiters exhibited lower rates of tooth decay. The variety of results may be due to many factors, including sample sizes verified in some studies, methodology, and the multifactorial etiology of caries (34, 39, 40). The majority of dentists who responded to our survey also incorrectly identified caries as a characteristic intraoral symptom of AN (61.0%). Only 3.8% of dentists answered correctly by marking “unsure - among researchers, there are different opinions”. Our study confirms the previous research, which found that a large percentage of dental practitioners also mistakenly identified dental caries as manifestations of eating disorders (41, 42). Over time, poor oral hygiene may also increase the risk for periodontal disease (23). There are some controversies between the appearance of periodontitis and AN. Periodontal health in patients with AN may be compromised by their nutritional status. Avitaminosis, anemia, and chemical irritation associated with self-induced vomiting can exacerbate periodontal disease and contribute to the development of periodontitis (43). Chiba et al. (44) observed worse periodontal conditions in patients with AN and bulimia nervosa compared with the control group, whereas Touyz et al. (45) showed that both patients with AN and bulimia had changes indicative of gingivitis and gingival recession but not periodontitis. In our study, only 5.1% of dentists answered ”unsure–among researchers, there are different opinions“. However, two out of three respondents answered ”agree,“ 8.1% ”disagree“ and 22.5% ”don't know.“

Enamel erosion is the most common intraoral complication that occurs due to purging by vomiting and becomes apparent about 6 months after onset (13, 21). It is usually seen on the palatal side of the anterior maxillary teeth, as this side is exposed most directly to acid (32). This specific type of enamel erosion is termed perimylolysis and has a smooth, glossy appearance (16). In more severe cases, erosions occur on the palatal margin of the maxillary teeth, as well as on the mandibular molar occlusal surfaces (46). The surface and depth of changes are closely related with the duration of the disease; they can be significant, often extending to the deeper layers of the dentin and even reaching the pulp (36). Dental erosions in patients with AN of the purgative subtype were less common in areas protected by the tongue, buccal mucosa, or lips from direct acid exposure (46). In the case of restrictive AN, dental erosions are not often present (9). In those patients, erosions may occur on buccal or labial surfaces as a result of overconsumption of highly acidic foods, such as raw citrus fruits (which provide a low amount of calories) (9). In addition, regular consumption of low pH products may cause chronic oral acidity, exacerbating the tooth erosion caused by vomiting (46). Sometimes, perimylolysis is the only oral manifestation apparent in patients who induce vomiting (9, 21). In the present study, most dentists correctly identified the loss of tooth structures caused by erosions. Usually, dental erosion leads to the emergence of dentin hypersensitivity (9, 10). Patients may then complain of short and sharp pain arising from exposed dentin in response to chemical, thermal, tactile, or osmotic stimuli (9, 10, 13). In this study, 71.6% of dentists were aware of tooth hypersensitivity in patients diagnosed with AN. Our results confirm that a large percentage of dentists correctly identified erosions and dentin hypersensitivity as signs of AN with a vomiting component (22, 41).

The present study assessed various factors on knowledge. There were no differences between men and women, and the activity of dentists (number of patients admitted per week) did not affect the knowledge about AN. However, the level of knowledge was influenced by the age and clinical experience of responders. Older dentists with many years of clinical experience gained better knowledge than the younger dentists who completed their studies 4–10 years ago. These differences can be explained by greater clinical experience and by the deepening of knowledge and desire as a result of specialization. This, moreover, confirms the comparisons of the results of general practitioners and specialists (14.8 vs. 16.3, *p* < 0.05). It seems that the exploration of knowledge resulting from the specialization of dentists is also responsible for the much worse knowledge of dentists working in the countryside as compared to those in large cities, where specialists have their dental practices.

The current survey also asked dentists to assess their knowledge about AN. Most dentists rated their knowledge about AN to be sufficient to good (84.3%), and one in 10 dentists rated their knowledge as poor. The results differ from those registered by Swedish and Norwegian dentists who perceived their knowledge as relatively and good as appropriate at 64.6% (Swedish) and 74.3% (Norwegian), and as poor in every fourth Swede and almost every third Norwegian. This suggests good self-esteem on behalf of Polish doctors. Additionally, comparing their self-esteem with the results obtained in the text definitely allows us to state that Polish dentists are aware of their knowledge about AN. Persons who assessed their knowledge as poor obtained significantly fewer points than those who considered their knowledge good.

In the present study, the surveyed dentists, similar to those from Sweden and Norway (24, 25), gained knowledge about AN mainly from the media, self-education, and dental school. They acquired such information much less frequently during specialist courses, which may explain the dentists' poorer knowledge of oral manifestations.

## Limitations

The survey was distributed *via* e-mail and social media. In such cases, the responsiveness is low. Currently, due to the COVID-19 pandemic, the lack of conferences and other professional meetings during which a survey could be conducted, was the only applicable option. In the future, after a period of quarantine, when the conferences will be held again, we may consider conducting a survey among the participants of these meetings. Although the responsiveness will be greater, the respondents will be recruited only from among dentists interested in broadening their knowledge.

## Conclusions

There is no study regarding the Polish dental practitioner's knowledge of eating disorders. The results of this study indicated that the awareness about AN is sufficient in relation to the general symptoms of anorexia However, Polish dentists have difficulties with the oral symptoms of AN. Therefore, there is a need to increase continuing education in this field, which can improve early diagnosis of this disease by dental practitioners and referral to specialists for treatment.

## Data Availability Statement

The raw data supporting the conclusions of this article will be made available by the authors, without undue reservation.

## Ethics Statement

Ethical review and approval was not required for the study on human participants in accordance with the local legislation and institutional requirements. Written informed consent for participation was not required for this study in accordance with the national legislation and the institutional requirements.

## Author Contributions

AK-Z, KK, and ML contributed to conception and design of the study and wrote sections of manuscripts. EM and WD wrote the sections of manuscript. KS performed statistical analysis. All authors contributed to manuscript revision, read, and approved the submitted version.

## Funding

This research was conducted with funding from Pomeranian Medical University in Szczecin.

## Conflict of Interest

The authors declare that the research was conducted in the absence of any commercial or financial relationships that could be construed as a potential conflict of interest.

## Publisher's Note

All claims expressed in this article are solely those of the authors and do not necessarily represent those of their affiliated organizations, or those of the publisher, the editors and the reviewers. Any product that may be evaluated in this article, or claim that may be made by its manufacturer, is not guaranteed or endorsed by the publisher.
